# Anorexia Nervosa and Psychiatric Comorbidities – It’s not all about food

**DOI:** 10.1192/j.eurpsy.2023.1106

**Published:** 2023-07-19

**Authors:** A. S. Morais, F. Martins, P. Casimiro, V. Henriques, N. Descalço, R. Diniz Gomes, S. Cruz, N. Costa

**Affiliations:** Psychiatry, Hospital Garcia de Orta, Lisbon, Portugal

## Abstract

**Introduction:**

Anorexia nervosa (AN) is a severe psychiatric disorder that usually begins during adolescence and is associated with a high risk of mortality and morbidity, its treatment is complex and often ineffective. Psychiatric comorbidity is common in patients with eating disorders (with the prevalence of 20–95%), namely 39% in AN.

**Objectives:**

The purpose of the authors is to review the most common areas of psychiatric comorbidity in AN, how it affects the course of both diseases and the potential treatment approaches.

**Methods:**

A brief non-systematized review is presented, using the literature available on PubMed and Google Scholar.

**Results:**

The most common psychiatric comorbidities in AN are: Affective disorders in 24-38% (mainly unipolar depression which can appear in up to 75% of patients, compared to 11% in bipolar disorder); Anxiety disorders in 25.5% (11% with panic disorder, 20% social phobia/social anxiety disorder, 15% specific phobias, 10% generalized anxiety disorder, 13% post-traumatic stress disorder); Obsessive compulsive disorder in 12%; Substance use disorders at 17%; Personality disorders around 30%. Other pathologies occur less commonly but can have a significant impact on the patient, namely Autism spectrum disorder (predictive factor for unfavourable outcome) or Schizophrenia (there are reports of reciprocal relationships between the two pathologies).

Some of these comorbidities may increase mortality in AN, namely unipolar depression, personality disorders, alcohol and illicit drug use.

The profound impact that starvation has on mood and cognition is well known. It can condition symptoms that are confused with other psychiatric diseases and change their clinical presentation. As such, the specific clinical characteristics and the therapeutic approach will be presented for each of the psychiatric comorbidities.

**Conclusions:**

Early diagnosis and treatment of psychiatric comorbidities in AN are essential to improve the prognosis of this eating disorder. The additional treatment of these pathologies will increase complexity of the already challenging treatment of AN, with the additional symptomatology often being perpetuated by an uncontrolled eating disorder and a poor compliance to treatment.

The limited evidence available for approaching these cases is based on the few studies available, most with insufficient samples.

**Disclosure of Interest:**

None Declared

